# Harnessing Energy of a Treadmill for Push-Off Assistance During Walking: *In-Silico* Feasibility Study

**DOI:** 10.3389/fbioe.2022.832087

**Published:** 2022-02-16

**Authors:** Matej Tomc, Zlatko Matjačić

**Affiliations:** ^1^ Research and Development Unit, University Rehabilitation Institute Republic of Slovenia, Ljubljana, Slovenia; ^2^ Laboratory of Robotics, Faculty of Electrical Engineering, University of Ljubljana, Ljubljana, Slovenia

**Keywords:** human locomotion, rehabilitation, ankle exoskeleton, timing, elastic tendon

## Abstract

Regaining efficient push-off is a crucial step in restitution of walking ability in impaired individuals. Inspired by the elastic nature of ankle plantarflexor muscle-tendon complex, we propose a novel rehabilitation device: Ankle Exoskeleton using Treadmill Actuation for Push-off assistance (AN-EXTRA-Push). Using a brake and an elastic tendon, it harnesses energy of a moving treadmill during stance phase, then releases it during push-off to aid with plantarflexion torque generation. We studied the feasibility of such a device and explored some key design and control parameters. A parameter sweep of three key parameters (brake engagement timing, brake disengagement timing and elastic tendon stiffness) was conducted *in-silico*. Results suggest that such a device is feasible and might inherently possess some features that simplify its control. Brake engagement timing and elastic tendon stiffness values determine the level of exoskeleton assistance. Our study affirms that timing of assistive torque is crucial, especially the timing of assistance termination which is determined by brake disengagement timing. Insights acquired by this study should serve as a basis for designing an experimental device and conducting studies on effects of AN-EXTRA-Push in humans.

## Introduction

Walking is the primary mode of human locomotion. A major contributor to walking capacity is the ability of the ankle-foot complex to produce positive work, accelerating the body’s center of mass and facilitating step to step transitions (Beyaert et al., 2015; Awad et al., 2020b). In healthy human gait the ankle joint produces the highest amount of positive power among all the joints while also being the most metabolically efficient source of work needed for propulsion (Farris and Sawicki, 2012). The majority of power is produced in a burst late in the stance phase termed push-off (Huang et al., 2015).

Individuals suffering from neuromuscular impairments (e.g. post stroke) have reduced propulsion generating capabilities due to muscle weakness and loss of voluntary muscle control and coordination (Beyaert et al., 2015; Li, 2017). Modern clinical rehabilitation programs often include robot assisted rehabilitation in which treadmills and/or exoskeletons are used for gait rehabilitation purposes. Despite the importance of regaining efficient push-off for restitution of healthy gait, most modern exoskeletons used in clinical practice are neglecting the ankle joint or focusing just on dorsiflexion needed for toe clearance during swing phase (Westlake and Patten, 2009; Kim et al., 2015).

In recent years a new research field of ankle exoskeletons focused specifically on the gait subtask of propulsion has emerged. The common feature of these exoskeletons is that they provide positive mechanical work at the ankle joint, thereby reducing the demand for biologically produced work and ideally lowering the metabolic cost of transport for the wearer. Ankle exoskeletons can be classified as either passive or active. Passive ankle exoskeletons (Collins et al., 2015; Wang et al., 2020) store energy in elastic structures during early parts of stance phase and release it during push-off. Since there are no power supplies and electronics, these devices are extremely lightweight, but their controllability is limited. Active ankle exoskeletons use an external energy source to produce torque at the ankle joint. Pneumatics (Sawicki and Ferris, 2008; Takahashi et al., 2015) and electrical motors (Wu et al., 2011; Mooney et al., 2014; Awad et al., 2020a) have been used to actuate the ankle exoskeletons. A supply of compressed air is needed to use a pneumatic actuator, limiting the use of these exoskeletons to treadmill walking. Exoskeletons with electrical motors can either be tethered (Wu et al., 2011; Witte et al., 2015) with power supplies and motors mounted on the side of the treadmill or portable, in which case a battery pack is worn by the user (Mooney et al., 2014; Asbeck et al., 2015; Awad et al., 2020a). In both cases Bowden cables have been used to transfer the power from the source to the ankle joint. This is an effective way of transmitting power from a distant power supply to the ankle while also imposing weaker restraints on the degrees of freedom of the ankle compared to direct drive mechanisms. The main drawback of using Bowden cables is that they are subject to highly nonlinear friction forces, which makes the transmission variable and proper control challenging (Grosu et al., 2019). Both rigid orthoses (Mooney et al., 2014; Collins et al., 2015; Takahashi et al., 2015; Witte et al., 2015) as well as soft textile-based interfaces (Asbeck et al., 2015; Awad et al., 2020a) have been proposed. The advantage of soft textile-based interfaces is that problems with joint misalignments are avoided, but relatively small forces can be applied on the ankle and foot, which might not be sufficient to offset the negative impact of the added mass worn by the user (Asbeck et al., 2015). The challenge of reducing external mass added to the body is present across all types of exoskeletons. The problem of added mass is exacerbated in ankle exoskeletons due to the distal position of the ankle joint which leads to a significant increase in the segments’ inertia (Browning et al., 2007). To be comfortably worn, the weight of the device should not exceed 2% of the user’s body mass (Soule and Goldman, 1969; Meuleman et al., 2013). In case of portable active ankle exoskeletons, this goal is most often not achieved (Moltedo et al., 2018).

An untapped readily available source of energy that could potentially be used to power an ankle exoskeleton is a treadmill. Treadmills are already parts of clinical rehabilitation setups and are necessary for any tethered ankle exoskeleton’s operation. Inspired by the elastic nature of the ankle plantarflexor muscle-tendon complex (Alexander, 1989; Ishikawa et al., 2005; Kharazi et al., 2021; Krupenevich et al., 2021), we propose a novel ankle exoskeleton design, that aims to combine the best features of passive and active ankle exoskeletons.

The device we propose is called Ankle Exoskeleton using Treadmill Actuation for Push-off assistance (AN-EXTRA-Push). It operates by harnessing energy of a moving treadmill in elastic structures during stance phase, then releasing it during push-off to aid with plantarflexion torque generation. In this paper, we present an *in-silico* study that investigates the feasibility of our novel approach and gives insights into key design and control parameters of AN-EXTRA-Push.

## Materials and Methods

### AN-EXTRA-Push Concept

**FIGURE 1:**
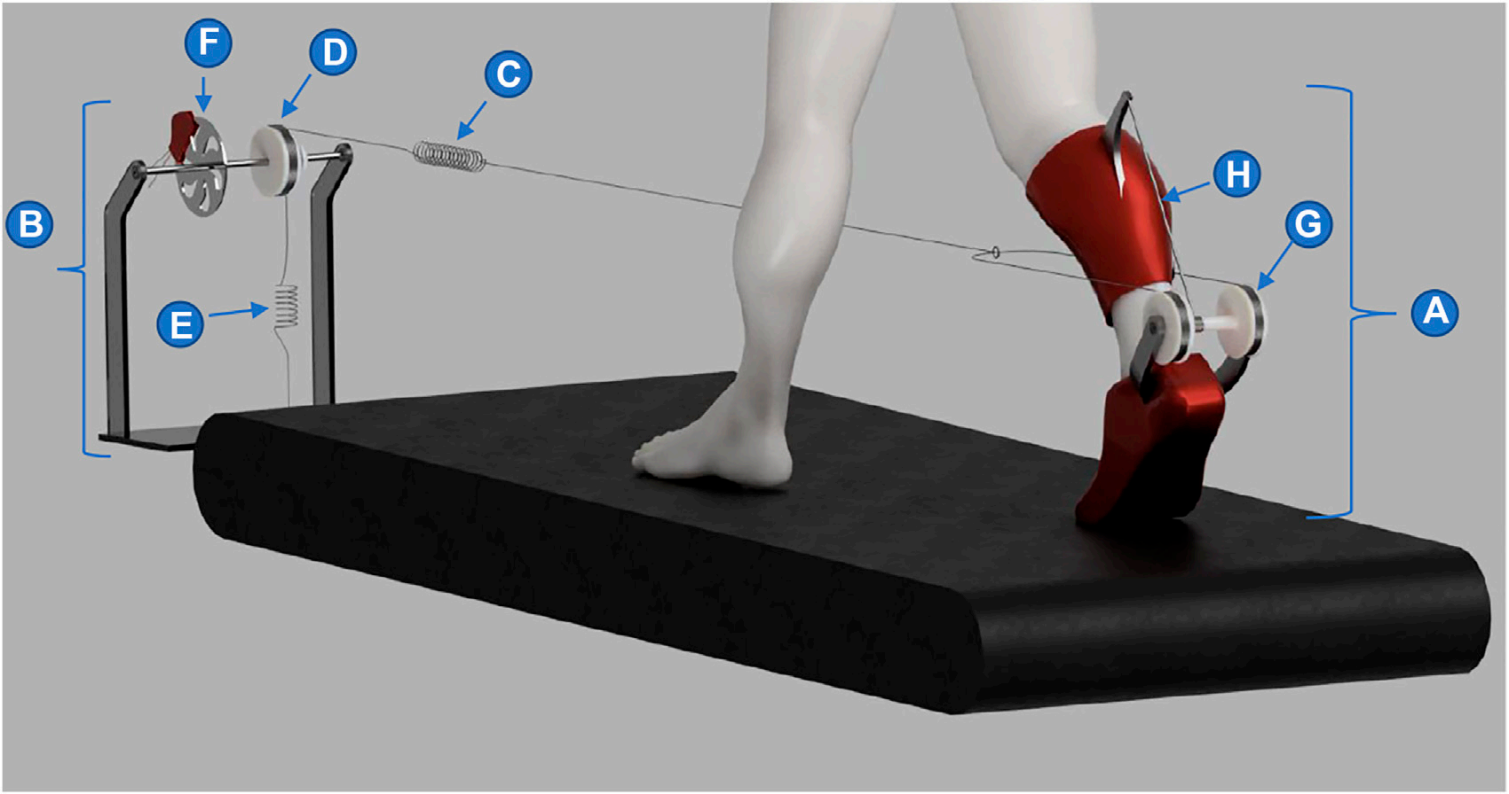
AN-EXTRA-Push mechanical design. A lightweight orthosis (a) is worn by the user. It is connected to a braking and pretentioning module (b) using an elastic tendon (c). The braking and pretensioning module consists of a pulley (d), a constant force spring (e) and a brake (f). On the foot part of the orthosis a three spool structure (g) is mounted. It connects the elastic tednon (c) with a rigid tendon (h) attached to the shank part of the orthosis.

AN-EXTRA-Push consists of an orthosis [element (A) in Figure 1] worn by the user, a braking and pretensioning module [element (B) in Figure 1] mounted in front of a treadmill, and an elastic tendon [element (C) in Figure 1] connecting the two. Similarly to other passive and tethered systems, no motors or batteries are worn by the user, thereby keeping the inertia added to the body at a minimum, far below the maximum recommended values (Soule and Goldman, 1969; Meuleman et al., 2013). The first end of the elastic tendon is connected through a pulley [element (D) in Figure 1] highly compliant constant force spring [element (E) in Figure 1]housed in the braking and pretensioning module. The purpose of the constant force spring is to prevent slacking of the elastic tendon. The axis of rotation of the pulley can be stopped by engaging the brake [element (F) in Figure 1], effectively fixing one end of the elastic tendon in place. The other end of the elastic tendon is split in two and attached to the outer spools of a three-spool structure [element (G) in Figure 1] that is part of the orthosis worn by the user. The three-spool structure consists of two outer spools and an inner spool with a radius five times smaller than the outer two. All three spools are affixed together and rotate synchronously about their common axis. Using handles with bearings the three-spool structure is attached to the foot segment with its only degree of freedom being the rotational axis of the spools. This rotational axis should be parallel to the ankle’s own plantarflexion/dorsiflexion axis. The inner spool of the three-spool structure is connected to a fixed attachment point on the shank segment of the orthosis using a rigid tendon [element (H) in Figure 1]. In function, the three-spool structure resembles a reduction drive. The ratio between the outer and inner spools determines the winding/unwinding speed ratio of the two tendons and the ratio between the forces present in the tendons.

**FIGURE 2:**
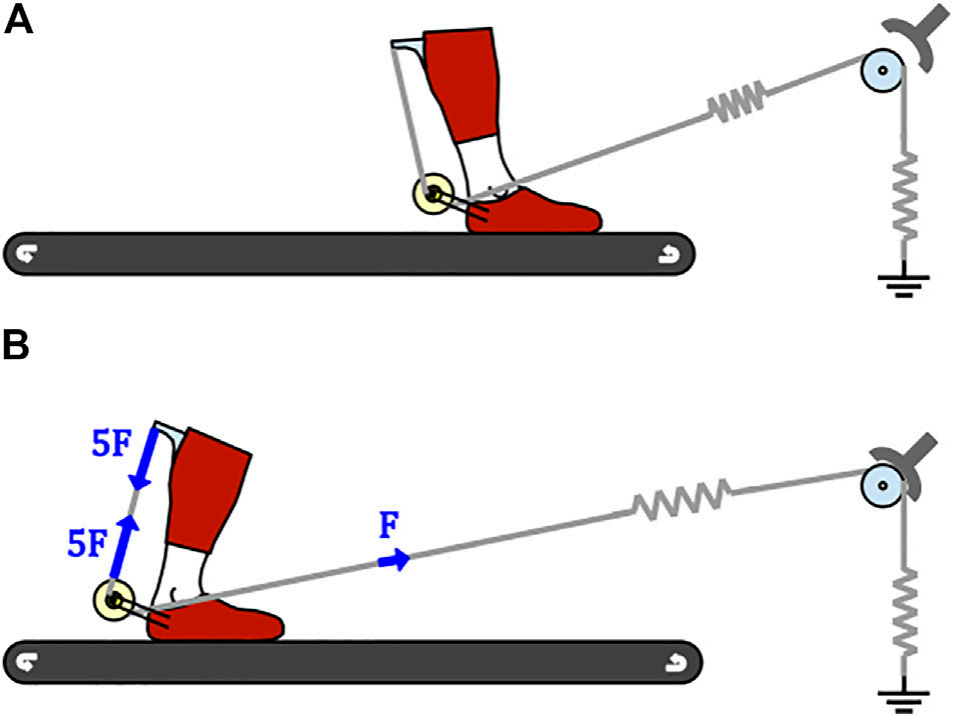
Schematic representation of AN-EXTRA-Push use. **(A)** AN-EXTRA-Push before the brake is engaged. **(B)** AN-EXTRA-Push at the moment of peak forces present in the system.

When the brake is engaged, the constant force spring ceases to have effect on the elastic tendon and the comparatively stiffer elastic tendon can start to stretch. This should be done in early to mid-stance (Figure 2A) after weight has been transferred to the leg with the exoskeleton. Provided that sufficient friction is present, the foot is pulled by the treadmill posteriorly, away from the braking and pretensioning module. Throughout the stance phase the ankle is dorsiflexing. Both effects demand the lengthening of the elastic tendon, which can only be achieved by increasing the length of the elastic structures and proportionally increasing the force produced by them. Close to the moment of heel-off, peak force is present in the system (Figure 2B). The force in the elastic tendon is pulling the foot in the anterior direction. Amplified by the ratio between the outer and inner spools, a five times greater force is present in the rigid tendon creating equal and opposite forces between the shank attachment point and the three-spool structure axis affixed to the foot. Since both of those are positioned posteriorly to the ankle, plantarflexion torque is generated in the ankle joint. After push-off is initiated, around the time when peak dorsiflexion is achieved, the brake should be disengaged. Ideally, most of the energy stored in the elastic tendon had already been used up for the push-off assistance up to that point. Any remaining energy may be dissipated using an appropriate damper added to the braking and pretensioning module.

The main parameters shaping the ankle torque assistance profile provided by AN-EXTRA-Push are elastic tendon stiffness and brake engagement and disengagement timings. Previous research has shown the importance of optimal assistance power and timing in ankle exoskeleton-based rehabilitation (Malcolm et al., 2013; Galle et al., 2017; Moltedo et al., 2018; Moltedo et al., 2020). Experimentally testing the entire possible parameter space would be a prohibitively time-consuming task. Thus, in this study, we utilized *in-silico* design space exploration to determine the ranges of feasible values for key parameters. These can then serve as a base for designing an experimental device and an a protocol for testing in humans.

### Modeling and Simulations

**FIGURE 3:**
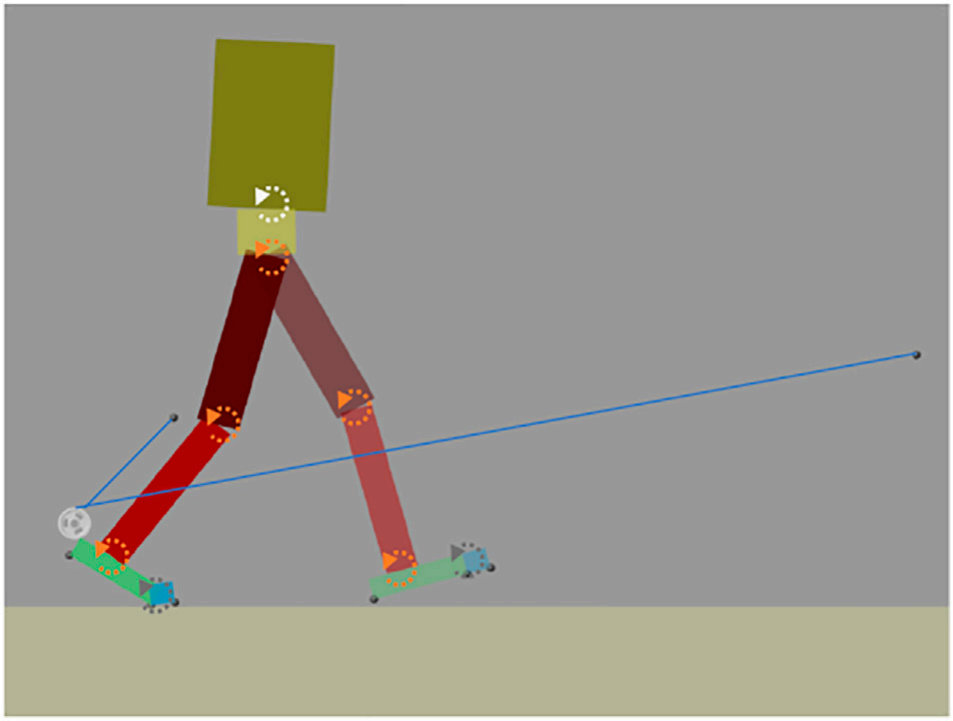
A 2D dynamic walking model for investigating the effects of AN-EXTRA-Push on gait. AN-EXTRA-Push is worn on the right leg—the trailing leg in the pricture. Arrows mark the positions of rotary joints. Gray arrows: passive metatarsophalanegal joints; white arrow: controlled but not optimized lumbo-sacral joint; orange arrows: joints subject to optimization. Hip joints for both legs overlap.

We developed a dynamic walking model and used it to investigate the effects of AN-EXTRA-Push system on gait kinematics and kinetics. All modeling and simulations were done in MATLAB and Simulink (MathWorks, Massachusetts, United States), making extensive use of the Simscape Multibody library. We decided on a 2D model constrained to the sagittal plane. The model consisted of 10 rigid segments (a head-arms-trunk segment, a pelvis segment and pairs of thigh, shank, foot and toes segments) connected by rotary joints as shown in Figure 3. Inertial properties of the segments were chosen to closely resemble the inertial properties of a specific subject from a public gait database (Lund et al., 2018). The inertial parameters are shown in Supplementary Table S2 in Supplementary Material. The model was driven by direct application of torques to the corresponding joints. Exceptions to that are the metatarsophalangeal joints connecting the foot and toes segments, which were modeled as passive rotary springs. Geometry of the foot-ground contact was described by contact spheres under the heels, the metatarsophalangeal joints and the toe-tips (Owaki et al., 2021). When these spheres penetrated the plane representing the treadmill, normal and friction forces were calculated. Foot-ground contact model parameters are detailed in Supplementary Table S3 in Supplementary Material.

A simplified AN-EXTRA-Push was modeled. The elastic tendon was modeled as an ideal unilateral spring. Forces created by the constant force spring and transient phenomena after brake engagements/disengagements were not considered. Modeled inertiae of the shank and foot segments of the right leg were adjusted to account for an estimated total of 0.8 kg of additional mass representing the orthosis.

Gait kinematics from a public dataset of healthy human walking (Lund et al., 2018) were used as a base for control of the model. A gait cycle lasted 1.21 s and the walking speed was 1.18 m/s. The trajectories were manually adjusted to create a stable symmetrical gait cycle. For the purposes of our simulations, four of these identical gait cycles were chained together. Local PID controllers at each joint were used to produce joint torques that made the joints follow their respective reference kinematics. These torques were supposed to simulate the body’s own torques needed to produce the desired kinematics and were therefore referred to as biological torques. A diagram of the simulation scheme is shown in Supplementary Figure S1 in the Supplementary Material.

First, normal gait without the exoskeleton (referred to as NO-EXO) was simulated. A stable and physiologically adequate gait pattern was produced. Then AN-EXTRA-Push model was added to the walking model. When forces and torques were exerted by AN-EXTRA-Push onto the body, a slight adjustment of reference kinematics was necessitated. An optimization problem with the goal of creating new reference kinematics for the hip, knee and ankle joints that resulted in stable and physiological gait was formulated. The lumbo-sacral joint was not included in the optimization. Rather, a simple feedback loop that ensured the upright position of the HAT segment was implemented. Genetic algorithm was used to solve the optimization problem. Reference kinematics of the NO-EXO condition were used as the initial guess. The fitness function consisted of a weighted sum of five criteria: time until the fall, distance traveled compared to NO-EXO condition, ground reaction force impulse at heel-strike, total absolute joint power and similarity of torque profiles to NO-EXO condition (equations S1-S6 in Supplementary material). Solution-space boundaries had been set to ensure that the optimal solution stayed close to the kinematics of NO-EXO condition. The process of solution-space discretization and reduction as well as fitness function formulation is detailed in the Supplementary Material.

A parameter sweep analysis was performed. Permutations of eight elastic tendon stiffness values, seven brake engagement timings and 11 brake disengagement timings were tested. The values of the parameters are shown in Table 1. Elastic tendon stiffness values are normalized to body mass. For each triplet of parameters, the optimization problem was solved. Due to the heuristic nature of the optimization method and high nonlinearity of the model, some uncertainty was present in the solution. Because of this, the optimization was repeated four times for each combination of parameters. Out of the four solutions, one was discarded as an outlier. Out of the remaining three, the one with the median joint torque characteristic was chosen as a representative solution.

**TABLE 1:** Parameter sweep analysis values. One parameter value from each column is chosen to form a parameter triplet. All possible triplets were tested.

Elastic tendon stifness [N/m/kg]	Brake engagement timing [stride %]	Brake disengagement timing [stride %]
0.91	20	44
1.52	23	47
2.12	26	50
2.73	29	51
3.79	32	52
4.85	35	53
6.97	38	54
9.09		55
		56
		59
		62

Hip, knee and ankle angles, biological torques and biological powers were analyzed and compared between different parameter combinations of the parameter sweeps. Torques, powers, ground reaction forces and elastic tendon forces of AN-EXTRA-Push were normalized to body mass. Our main criterion for push-off assistance efficacy was reduction of positive ankle work. Positive ankle work was calculated as an integral of positive ankle power between 40% and 60% of stride. The percentage of reduction was calculated with respect to the NO-EXO condition. Where AN-EXTRA-Push use would require additional negative power compared to NO-EXO condition, those parts were not considered as adequate assistance and were not included in positive ankle work reduction calculation. Using Pearson’s correlation coefficient, linear correlation between the parameter from the parameter sweep and the positive ankle work reduction was calculated.

## Results

**FIGURE 4:**
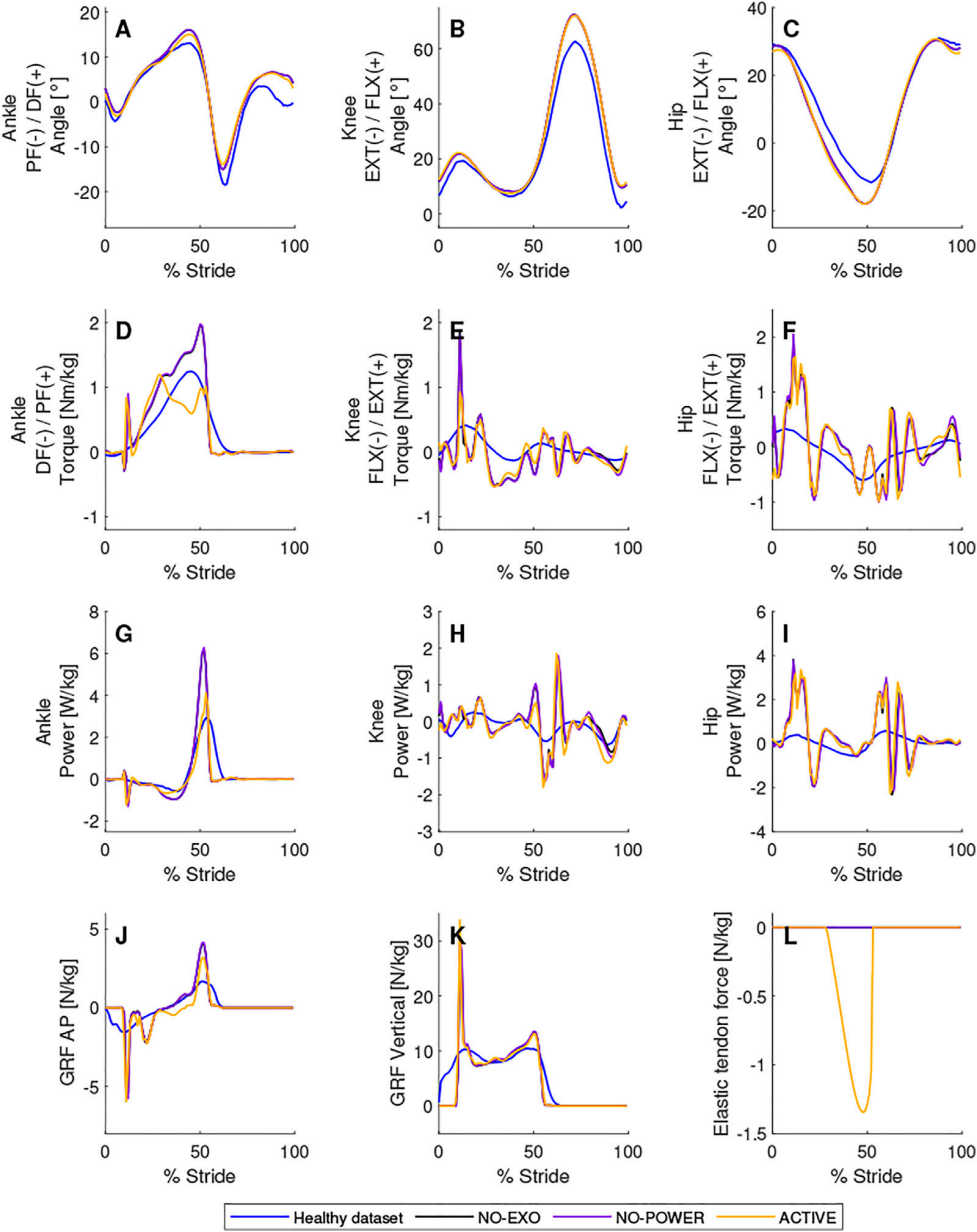
Comparison between a dataset of healthy people and modeled walking with an active, unpowered and without AN-EXTRA-Push exoskeleton. Joint angles **(A–C)**, biological torques **(D–F)** and powers **(G–I)** are shown for the right leg on which AN-EXTRA-Push is worn. In **(J)** and **(K)**, antero-posterior and vertical ground reaction forces are shown. In **(L)**, force in the elastic tendon is shown. Conditions are abbreviated and color coded as follows: healthy subjects average from a public database (Healthy dataset) (blue), without exoskeleton (NO-EXO) (black), unpowered exoskeleton (NO-POWER) (purple), and a powered exoskeleton condition with parameters: brake engagement timing of 29% of stride, brake disengagement timing of 53% of stride and elastic tendon stiffness of 4.85 N/m/kg (ACTIVE) (yellow).

**FIGURE 5:**
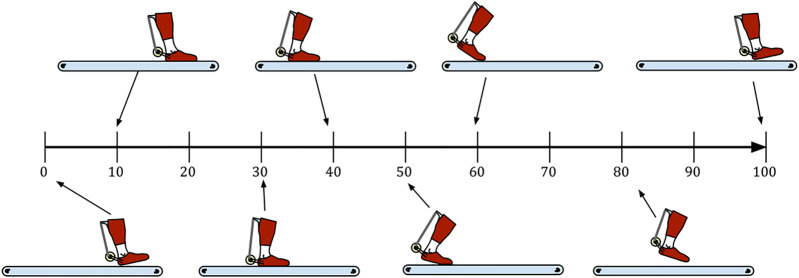
Timeline of gait events as observed during the ACTIVE condition. The leg reaches its most anterior position at 0% of stride. An almost flat-foot heel strike occurs around 10% of stride. The leg is moved posteriorly by the treadmill. Shortly after 50% of stride the heel leaves the treadmill, followed by toe-off event shortly before 60% of stride. The remaining part of stride consists of the swing phase.

**FIGURE 6:**
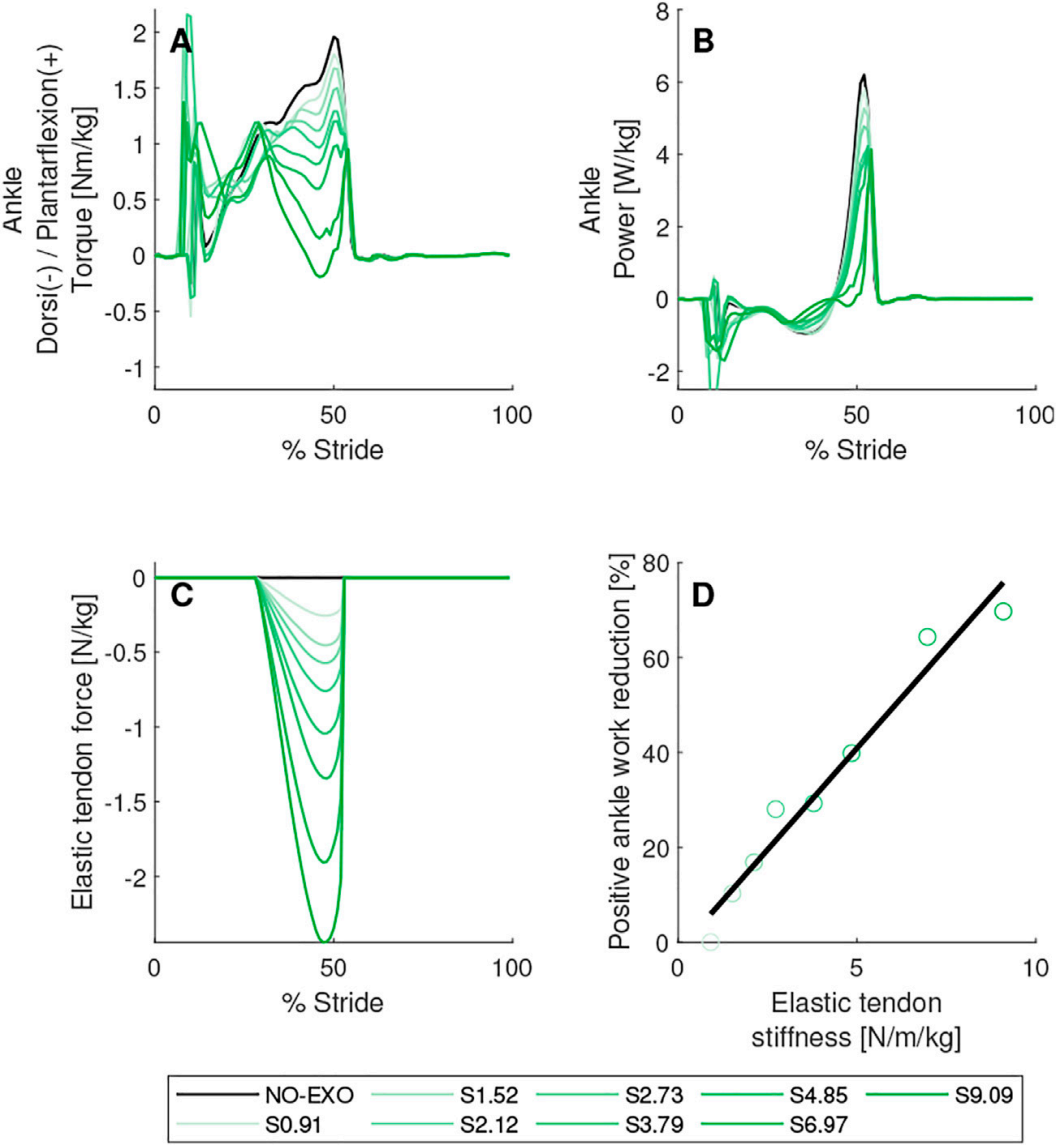
Elastic tendon stiffness parameter sweep. Biological torques **(A)** and powers **(B)** are shown for the right leg on which AN-EXTRA-Push is worn. In **(C)**, force in the elastic tendon is shown. In **(D)**, linear regression between elastic tendon stiffness value and positive ankle work reduction is shown. Brake engagement timing was set to 29% of stride and brake disengagement timing was set to 53% of stride. Conditions are abbreviated and color coded as follows: without exoskeleton (NO-EXO) (black) and active exoskeleton assistance with elastic tendon stiffness values of {0.91, 1.52 2.12, 2.73, 3.79, 4.85, 6.97, 9.09} N/m/kg (S0.91, S1.52 S2.12, S2.73, S3.79, S4.85, S6.97, S9.09) (green; from light to dark in the same order).

**FIGURE 7:**
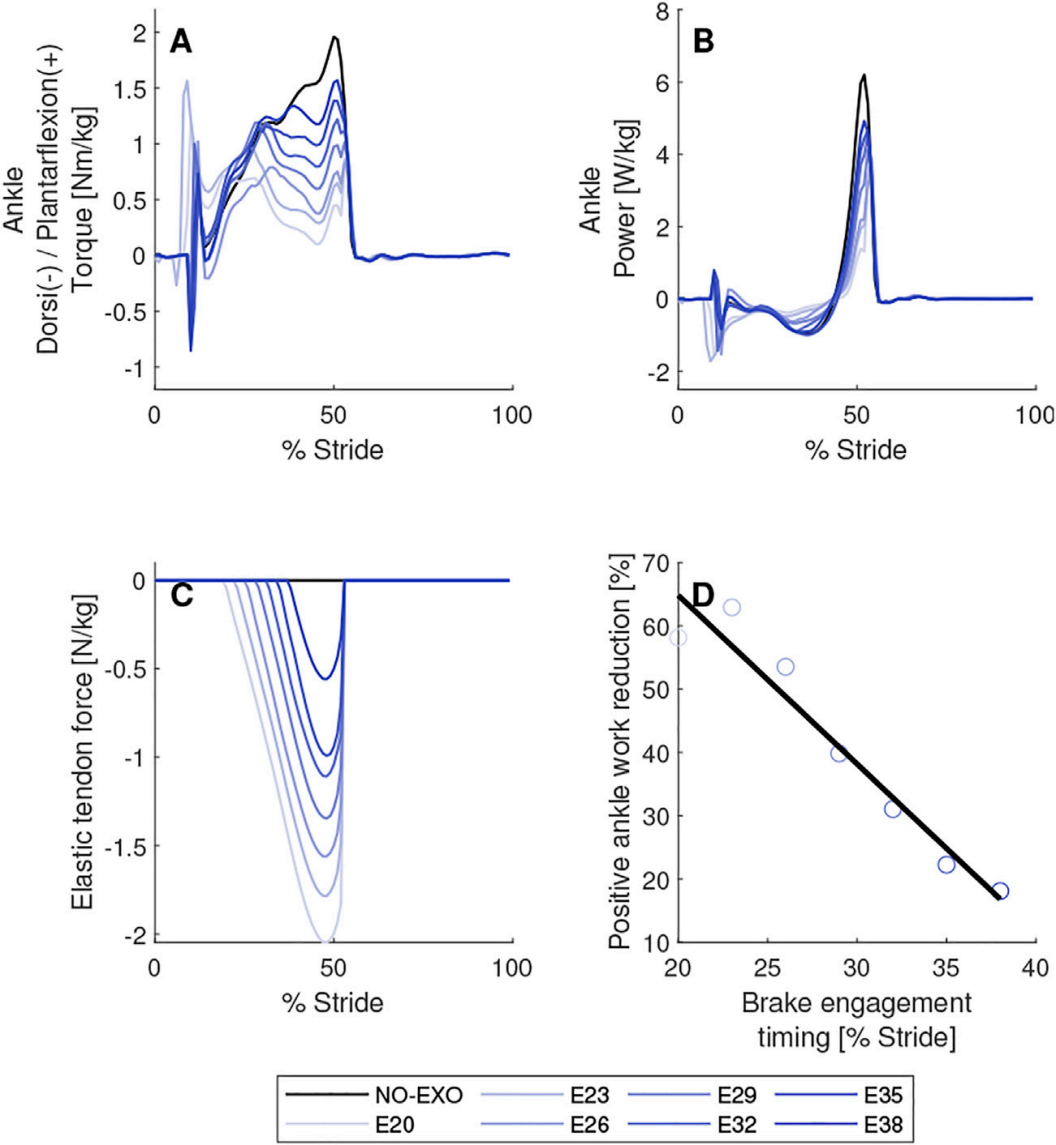
Brake engagement timing parameter sweep. Biological torques **(A)** and powers **(B)** are shown for the right leg on which AN-EXTRA-Push is worn. In **(C)**, force in the elastic tendon is shown. In **(D)**, linear regression between brake engagement timing and positive ankle work reduction is shown. Elastic tendon stiffness value was set to 4.85 N/m/kg and brake disengagement timing was set to 53% of stride. Conditions are abbreviated and color coded as follows: without exoskeleton (NO-EXO) (black) and active exoskeleton assistance with brake engagement timings of {20, 23, 26, 29, 32, 35, 38} % of stride (E20, E23, E26, E29, E32, E35, E38) (blue; from light to dark in the same order.).

**FIGURE 8:**
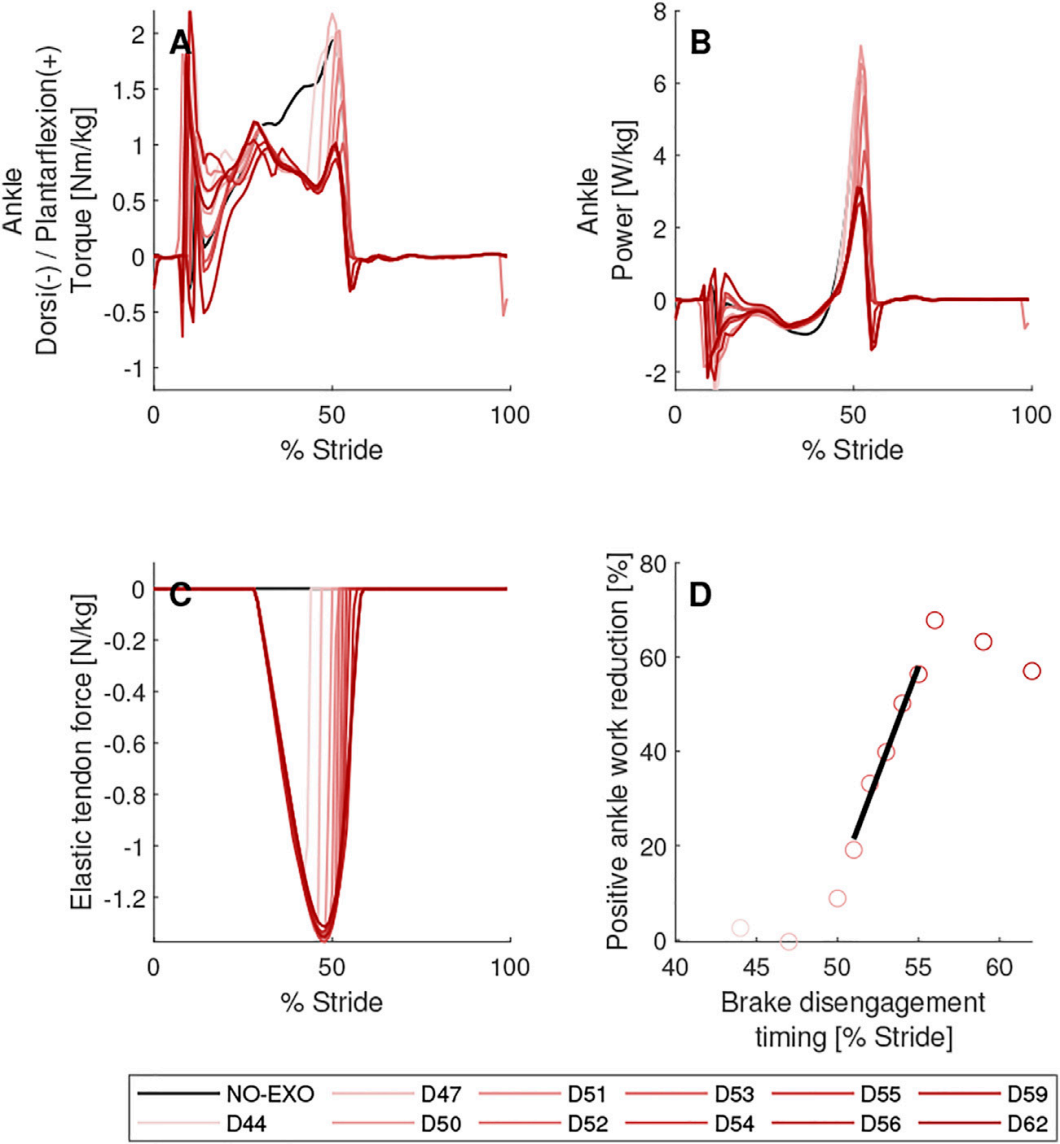
Brake disengagement timing parameter sweep. Biological torques **(A)** and powers **(B)** are shown for the right leg on which AN-EXTRA-Push is worn. In **(C)**, force in the elastic tendon is shown. In **(D)**, linear regression between brake disengagement timing and positive ankle work reduction is shown. Elastic tendon stiffness value was set to 4.85 N/m/kg and brake engagement timing was set to 29% of stride. Conditions are abbreviated and color coded as follows: without exoskeleton (NO-EXO) (black) and active exoskeleton assistance with brake disengagement timings of {44, 47, 50, 51, 52, 53, 54, 55, 56, 59, 62} % of stride (D44, D47, D50, D51, D52, D53, D54, D55, D56, D59, D62) (red; from light to dark in the same order.).

The optimization problem was solved for a total of 616 permutations of parameter triplets. For the sake of brevity, only some representative parameter combinations are presented. In Figures 4–8, only the results for the right leg, to which AN-EXTRA-Push was attached, are shown. Across all conditions almost no changes were observed in kinematics or kinetics of the contralateral leg (Spplementary Figure S2).

As shown in Figure 4, the ankle, knee and hip kinematics of the model were similar to those of healthy subjects. The 0% on the abscissa corresponds to the heel-strike event in normal walking. In the modeled gaits, the heel strike occurred at 10% of the gait cycle, which resulted in a spike in the vertical and antero-posterior ground reaction force that was then translated into a spike in the biological torques necessary to keep following the reference kinematics. The heel strike of the contralateral leg occurred at 60% of the stride. The effects of the contralateral leg heel strike manifested as fluctuations in the hip and knee torques, but not in the ankle torque.

Attaching the mass of AN-EXTRA-Push onto the leg resulted in negligible changes, as shown in an almost complete overlap between the unpowered (NO-POWER) and absent (NO-EXO) condition of AN-EXTRA-Push in Figure 4A–L.

To produce an example of AN-EXTRA-Push assisted walking (ACTIVE condition in Figure 4), the parameter triplet of brake engagement timing of 29% of stride, brake disengagement timing of 53% of stride and the elastic tendon stiffness of 4.85 N/m/kg was used. Minimal adjustments to the kinematics were necessary compared to the NO-EXO condition (Figures 4A–C). A sizable reduction in the biological ankle torque needed to produce the reference kinematics was observed (Figure 4D), while almost no changes were seen in the knee and hip torque requirements (Figures 4E,F). In the example, 41% of the positive work at the ankle joint needed for push-off was provided by AN-EXTRA-Push (Figure 4G). A video of the ACTIVE condition walking is attached in the Supplementary Material (Supplementary Video S1). For quick reference, leg positions in some key moments are shown in Figure 5.

### Elastic Tendon Stiffness Parameter Sweep

In Figure 6 the results of the elastic tendon stiffness parameter sweep are shown. In these simulations brake engagement and disengagement timings were set to 29% of stride and 53% of stride respectively. Eight elastic tendon stiffness values were tested, the lowest being 0.91 N/m/kg and the highest being 9.09 N/m/kg. Note that the distribution of tested values was not equidistant.

A reduction in positive biological ankle torque and power needed for push-off was observed across all experiments with the active assistance of AN-EXTRA-Push. In the example shown in Figure 6 the positive ankle work required for push-off was reduced by 5%–75% depending on the elastic tendon stiffness value. A strong linear correlation between the stiffness value and positive ankle work reduction was observed (*R* ^2^ = 0.96, *p* = 1.74*10^–5^). Joint kinematics remained almost unchanged across the entire sweep. A detailed figure of the parameter sweep results that includes graphs of joint angles, torques, powers and ground reaction forces can be found in the Supplementary material (Supplementary Figure S3).

### Brake Engagement Timing Parameter Sweep

In Figure 7 the results of the brake engagement timing parameter sweep are shown. In these simulations brake disengagement timing was set to 53% of stride and elastic tendon stiffness value of 4.85 N/m/kg was used. Brake engagement timings ranging from 20% to 38% of stride were tested.

A reduction in positive biological ankle torque and power needed for push-off was observed across all experiments with the active assistance of AN-EXTRA-Push. In the example shown in Figure 7 the positive work required for push-off was reduced by 20%–59% depending on the brake engagement timing. A strong negative linear correlation between the brake engagement timing and positive ankle work reduction was observed (*R* ^2^ = 0.94, *p* = 3.18*10^–4^). The earlier the brake was engaged, the more energy was stored and subsequently released by the elastic tendon. Joint kinematics remained almost unchanged across the entire sweep. A detailed figure of the parameter sweep that includes graphs of joint angles, torques, powers and ground reaction forces can be found in the Supplementary material (Supplementary Figure S4).

### Brake Disengagement Timing Parameter Sweep

In Figure 8 results of the brake engagement timing parameter sweep are shown. In these simulations brake engagement timing was set to 29% of stride and elastic tendon stiffness value of 4.85 N/m/kg was used. Brake disengagement timings ranging from 44% to 62% of stride were tested.

Three distinct groups of simulations were identified based on the brake disengagement timings. If the brake was disengaged at 50% of the stride or earlier, the energy stored in the elastic tendon was lost before it could be used to assist the push-off. In cases of brake disengagement timings of 44% and 47%, less than 5% of the positive ankle work needed for push-off was saved compared to NO-EXO condition. Approximately 11% of positive ankle work needed for push-off was saved if the brake disengagement timing was at 50% of stride. These simulations formed the “premature” group. No peak biological ankle plantarflexion torque reduction was observed in this group. The brake disengagement timings from 51% to 55% of stride were considered “on-time”. Simulations with brake disengagement timings of 56% of stride and later formed the “overdue” group. In these simulations all or nearly all energy stored by the elastic tendon was returned to the leg. Even after push-off was completed, the force remaining in the elastic tendon exerted external plantarflexion moment onto the ankle joint, creating a need for biological dorsiflexion moment to prevent a change in kinematics. After all stored energy was spent and the swinging leg reached a position, where the elastic tendon was no longer elongated, further delays in brake disengagement timing ceased to have any effect. In our case, this happened after 58% of stride.

If the “on-time” group was considered alone, there was a strong linear correlation between the brake disengagement timing and positive ankle work reduction (*R* ^2^ = 0.98, *p* = 1.10*10^–3^). Within this group, positive ankle work also appeared to be more sensitive to the brake disengagement timing (trend line slope of 9.05) compared to the sensitivity to brake engagement timing (trend line slope of -2.63). A detailed figure of the parameter sweep that includes graphs of joint angles, torques, powers and ground reaction forces can be found in the Supplementary material (Supplementary Figure S5).

## Discussion

We explored the effects of using AN-EXTRA-Push–a novel proposed ankle exoskeleton, that harnesses treadmill energy for push-off assistance. The overall goal of the study was to investigate *in-silico* the feasibility of the novel approach and explore the appropriate ranges of key design and control parameters, which may serve as a basis for prototype device and practical experiment protocol design. Results of the simulation study indicate that the proposed approach is feasible. A wide range of feasible parameter combinations suggests that a personalized assistance profile could be provided to the user based on their needs.

### Interplay of Key Parameters

Increased elastic tendon stiffness values and earlier brake engagement timings both resulted in decreased biological torque and power needed for push-off. The positive biological ankle work needed for push-off can theoretically be reduced to zero (Zelik et al., 2014). In practice, the maximum assistive forces and torques are limited by the friction force between the foot and treadmill, since energy can only be harvested from the treadmill while it is in firm contact with the foot. Almost total elimination of biological work needed for push-off is however not desirable. Ankle exoskeletons are designed to work in parallel with the plantarflexor muscle-tendon complex. Other studies have shown that high amounts of exoskeleton power did not result in a proportional decrease of biological work (Mooney et al., 2014; Zelik et al., 2014; Collins et al., 2015; Jackson and Collins, 2015; Nuckols et al., 2020). Exoskeleton torques shown to reduce metabolic energy cost of walking rarely if ever exceeded 50% of biological ankle moment (Shafer et al., 2021). Higher amounts of exoskeleton assistance were shown to induce increased m. tibialis anterior activity (Galle et al., 2017), which suggests that humans do not fully rely on exoskeleton assistance and seek additional stabilization of the ankle joint by co-contracting ankle plantarflexor and dorsiflexor muscles. Our model lacks the capacity to predict such behavior. Multiple simulation results showing biological torque and power reductions greater than the literature recommended maximum values imply that AN-EXTRA-Push system has the capacity to provide more than enough assistive power before reaching the limits originating from its design.

Healthy humans make use of the elastic properties of the plantarflexor muscle-tendon complex to store energy during the early stance phase and then rapidly release the energy in late stance to create a short burst of propulsive power necessary for push-off (Alexander, 1989; Ishikawa et al., 2005; Krupenevich et al., 2021). In our simulations, the timing interval of positive ankle power encompasses only 13% of stride (from 43% to 56%). For synergistic activity of the ankle exoskeleton and the plantarflexor muscle-tendon complex, proper timing of the assistance is paramount (Malcolm et al., 2013; Galle et al., 2017; Moltedo et al., 2018; Moltedo et al., 2020). Temporal parameters contributing to AN-EXTRA-Push assistance timing are brake engagement and disengagement timing. Brake engagement timing marks the start of energy storage process and therefore directly influences the level of assistance. Results of the brake engagement timing parameter sweep show that for the parameter values tested, there was no change in timing of push-off initiation. We suspect that coupled with a high stiffness elastic tendon, an early enough brake engagement timing might result in a force high enough to provoke push-off in healthy humans. This situation should however not occur if the assistance forces and torques are kept under the maximum recommended values.

Results of brake disengagement timing parameter sweep suggest a narrower range of viable parameter values compared to the other two parameters. When the brake is disengaged, the assistance ceases almost immediately. In contrast to the brake engagement event, after which the elastic tendon begins to elongate and store energy, disengaging the brake causes a discontinuous change of the assistive forces and torques. Ideally the assistance should be shut off at the exact moment when the work for push-off is complete and no more assistive torque is required (Zhang et al., 2017). If the brake is disengaged prematurely, the assistance is cut off just before the peak of biological torque requirement. Apart from general lack of support at the critical time, the discontinuity in the assistance may also be seen as a perturbation. The model can respond to perturbations by instantly adjusting its biological torques, but a human cannot, which suggests that in practice, additional undesirable effects may follow such a perturbation. If the brake is disengaged too late, the exoskeleton will continue to provide plantarflexion torque to the ankle even after the requirements for it are no longer there. To counteract this, the model produced dorsiflexion torque in early swing phase. In practice, this may also cause a change in kinematics, where instead of opposing the exoskeleton torque, the person would plantarflex the ankle further, which may lead to problems with toe clearance during swing. In previous studies on ankle exoskeleton assistance timing (Malcolm et al., 2013; Galle et al., 2017; Moltedo et al., 2020), optimal timing of assistance termination was not investigated. Our results suggest that timing of assistance termination is key in ankle exoskeleton control and even small deviations from the proper assistance termination timing may be detrimental. Our findings are in accord with the results from a study by Zhang et al. (2017) in which rise time, fall time and peak assistance time were all considered and the intrapersonal variation of assistance termination timing was much smaller than the intrapersonal variation of timing of assistance initiation.

Effect of overdue brake disengagement is limited by the energy stored in the elastic tendon. After the heel loses contact with the treadmill and starts moving anteriorly and the ankle starts plantarflexing, the force in the tendon starts to decrease. Regardless of the brake disengagement timing, the leg reaches a position and configuration in which the elastic tendon is no longer elongated within approximately 5% of stride after push-off. Due to this, the effects from the overdue disengagement of the brake are inherently limited and any further delays have no additional undesirable effects. The effect also scales with the assistance level, so earlier brake engagement timings and higher elastic tendon stiffness values raise the importance of accurate brake disengagement timing.

### Simulation Study Assumptions

Our model is torque driven, two dimensional and constrained to the sagittal plane. It compares well to modern two-dimensional biorobotic models used in similar studies (Ji et al., 2021; Owaki et al., 2021) but does not reach the accuracy of complex 3D state-of-the-art models used for predictive human gait simulations (Nguyen et al., 2019; De Groote and Falisse, 2021). For the purposes of our simulation study, this is not a major limitation, as our focus was put on investigating the effects of AN-EXTRA-Push exoskeleton which is designed for assistance confined to the sagittal plane. In able bodied-gait, sagittal moments are the ones mainly responsible for propulsion (Sadeghi et al., 2001). We should therefore be able to capture both the key biological and exoskeleton torques as well as the interplay between them.

Large torque impulses usually not seen in human gait data are present at heel strikes. This is in part due to our implementation of the foot-ground contact and in part due to our decision not to use a low frequency lowpass filter on the data. The unfiltered data allows for better observations of instant effects of brake engagements and disengagements. The spikes created by the heel strikes seen in ground reaction forces and further reflected in all joint torques do not affect the part of the stride that is of interest to us. The impulsive heel strikes may also be a reason as to why the optimization favors making floor contact with the flat foot over striking with the heel and rolling over it. This behavior can be observed in the video in the Supplementary Material (Supplementary Video S1).

Within the optimization function a moderate preference towards gaits similar to NO-EXO condition was implemented. Deviations from both the reference kinematics as well as torques during intervals of no active assistance were discouraged. Our motivation in this was to constrain the search space of the genetic algorithm and speed up the optimization process. We also wanted to mainly explore the solutions where kinematics with and without the exoskeleton assistance remained similar. We consider this a fair assumption for healthy individuals with the capacity to adapt to the exoskeleton assistance, which has been confirmed by previous studies (Sawicki and Ferris, 2008; Galle et al., 2017).

All simulations were done at walking speed of 1.18 m/s. This speed was chosen because it is similar to walking speeds chosen by other researchers when doing experiments with healthy subjects (Sawicki and Ferris, 2008; Shafer et al., 2021). It is also close to the walking speed with minimal metabolic energy expenditure in healthy humans (Das Gupta et al., 2019).

The position of the braking and pretensioning module was chosen arbitrarily, based loosely on the expected practical implementation. It governs the static attachment point of the elastic tendon and therefore affects the direction and magnitude of the elastic tendon force. A gear ratio of 5:1 was chosen for the three-spool structure of AN-EXTRA-Push. This choice was made based on some preliminary simulations and is in essence also arbitrary. A lower gear ratio decreases the effect of the ankle angle on elastic tendon elongation. It also proportionally decreases the factor by which the force in the elastic tendon is amplified when transferred to the rigid tendon at the posterior side of the leg. The force in the rigid tendon acts on the foot and the shank in opposite directions and is therefore a pure source plantarflexion torque. The force in the elastic tendon mainly pulls the foot in the anterior direction. The sum of the force in the elastic tendon and the antero-posterior ground reaction force should always be lower than the friction force to prevent slips. A higher gear ratio would increase the effect of the ankle angle, but reduce the effect of the heel position on elastic tendon elongation, which would lead to more plantarflexion torque compared to the force pulling in the anterior direction, but would reduce the capability of AN-EXTRA-Push to harness the energy of a treadmill. Both the gear ratio and the position of the braking and pretensioning module can be seen as additional design parameters, that could be investigated with our model setup. We posit however, that the arbitrary values chosen were close enough to the optimal values, that the additional complexity of a simulation study with five parameters would not substantiate the trade-off of reduced granularity necessary for the completion of such a study.

### A Possible Approach to Implementation of Simulation Results

Exoskeletons that provide push-off assistance at the ankle joint do so in parallel with the body’s own plantarflexor muscle-tendon complex. To provide effective assistance, the external power generation of an exoskeleton must be synchronized with the body’s own efforts. Human-in-the-loop optimization is currently considered the state-of-the-art control method for assistive exoskeletons (Zhang et al., 2017). It requires variation of several torque parameters during several generations of optimization process to arrive at optimal values. This time-consuming and tedious process is unlikely to be implementable in application of exoskeletons with clinical populations. AN-EXTRA-Push may allow for simpler control strategies since many of the synchronization requirements are solved by its biomimetic design.

AN-EXTRA-Push mimics the behavior of the Achilles tendon. In humans, Achilles tendon is stretched over a longer period of time during the stance phase and then provides a burst of power during push-off (Alexander, 1989; Ishikawa et al., 2005; Kharazi et al., 2021; Krupenevich et al., 2021). An example of a biomimetic ankle exoskeleton working in parallel with the stretch and release of the Achilles tendon was developed by Collins et al. Collins et al. (2015). A spring and clutch mechanism was used to store the energy normally lost during the conversion from potential to kinetic energy of the center of mass during stance phase. The energy was then released during push-off, reducing the total energy cost of walking. AN-EXTRA-Push shares some similarities with this approach. During stance phase, part of the energy in the elastic tendon is stored from the same source as Collin’s exoskeleton. Predominantly however, AN-EXTRA-Push harvests the energy from the treadmill. Recoil of the elastic tendon is then initiated voluntarily by the walking subject by lifting their heel. The peak of assistance power is reached in synchrony with the biological needs. This synchrony is inherent in the design of AN-EXTRA-Push without any need for additional sensors or complex control strategies. It is likely, that such assistance may be more resistant to step-to-step variations, especially compared to predictive control strategies. Immediately after push-off completion, the assistance should cease. In terms of AN-EXTRA-Push, this requires timely brake disengagement. We intend to disengage the brake after a short delay following push-off initiation, the occurrence of which can be determined by measuring the elastic tendon force or elongation and ankle plantarflexion angle. The exact duration of the delay could be determined using Iterative learning control, which has previously been used in ankle exoskeleton assistance control (Zhang et al., 2015; Zhang and Collins, 2021). A similar control approach could also be used to determine the appropriate brake engagement timing. Finding the correct brake disengagement timing should be prioritized over the brake engagement timing.

After brake disengagement, the elastic tendon may still carry a considerable amount of energy. We foresee that the recoil of the elastic tendon caused by the release of the brake may excite oscillations in the elastic tendon, the pulley and constant force spring housed in the braking and pretensioning module. To prevent such behavior of the system, an active damping component to the braking and pretensioning module in the form of an electromagnetic eddy current brake could be used. With proper control of the electromagnet, we may ensure that the remaining energy from the elastic tendon is dissipated on the damper and no undesirable transient phenomena would occur.

## Conclusion

We propose a novel ankle exoskeleton AN-EXTRA-Push that operates by harnessing energy of a moving treadmill to assist with ankle plantarflexion torque generation. Using a brake and an elastic tendon, AN-EXTRA-Push stores the energy during stance phase and then returns it in the form of ankle power during rapid recoil of the elastic tendon during push-off. A parameter sweep of three key parameters (brake engagement timing, brake disengagement timing and elastic tendon stiffness) was conducted *in-silico*. Results of the simulation study confirm the feasibility of our approach. The power generation capacity of AN-EXTRA-Push exceeds the maximum assistance level that the humans are able to adapt to. Elastic tendon stiffness and brake engagement timing both contribute to the assistance level. Proper timing of the assistance initiation is triggered by the heel-rise event during subject’s gait and is inherent to the design of the exoskeleton. Timing of assistance termination is equal to brake disengagement timing. To avoid any undesirable effects, the brake should be disengaged during a narrow interval shortly after push-off completion. Of the three parameters explored, finding a proper brake disengagement timing should be prioritized. We propose that the findings of this study be used as a basis for designing an experimental device that will be used in the future to study the effects of AN-EXTRA-Push in humans.

## Data Availability

The raw data supporting the conclusion of this article will be made available by the authors, without undue reservation.
