# Supplementary material for: Harnessing Energy of a Treadmill for Push-Off Assistance During Walking: In-Silico Feasibility Study
Source: Front Bioeng Biotechnol. 2022 Feb 16;10:832087. doi: 10.3389/fbioe.2022.832087 (PMC8889039; doi:10.3389/fbioe.2022.832087)
Supplement: Supplementary file 1 [file DataSheet1.PDF]

Supplementary Material

1 DETAILS ON MODELING AND OPTIMIZATION

1.1 Optimization - solution space reduction

Our goal was to find joint angle trajectories which, when followed by our model, would resulted in a stable, physiological gait. We used a genetic algorithm to search for an optimal solution. We encoded our solution candidates into a 36 integer vector - six of the integers pertaining to each of hip, knee and joint angles of each leg. When appropriately scaled, these six integers represent the values of deviations from the joint angle trajectories of the NO-EXO condition at preset equidistant points of the gait cycle. Connecting these points with cubic splines and summing them with the trajectories from the NO-EXO condition creates new solution candidates.

The solution space was constrained so that no solutions that demanded hyperflexion or hyperextension of any joints were possible. Additionally, all solutions were constrained so that the resulting angle trajectories could not deviate more than 14 degrees from the NO-EXO condition. This eliminated any solutions where the model would adopt a completely different gait pattern, as we were interested in effects of AN-EXTRA-Push on healthy subjects that after the adaptation period, where the gait pattern should be similar to NO-EXO condition. This constraint also greatly reduced the solution space and sped up the optimization process.

1.2 Optimization fitness function

We used a multi-objective fitness function describing trade-offs between five performance criteria (Equation S1).

$$f_c = -w_1 * f_1 + w_2 * f_2 + w_3 * f_3 + w_4 * f_4 + w_5 * f_5 \tag{S1}$$

The highest absolute weight w_1 was assigned to the criterion pertaining to the duration of simulation. A simulation was stopped after a fall or after four completed gait cycles $4T$.

$$f_1 = \min(4T, t_{SimEnd}), \tag{S2}$$

The second criterion is the distance traveled by the model. The COM position of the model should stay as close as possible to the COM position of the model during NO-EXO condition simulation:

$$f_2 = \frac{1}{t_{SimEnd}} \int_0^{t_{SimEnd}} |X_{COM} - X_{COMref}|\, dt\,. \tag{S3}$$

With the third criterion we aim to minimize the impulse generated by the ground reaction force. The impulse energy is approximated by an integral of vertical component of the ground reaction force f_z that exceeds a threshold value f_{thresh}:

$$f_3 = \int_0^{t_{SimEnd}} \begin{cases} f_z\, dt & ; \quad f_z > f_{thresh} \\ 0 & ; \quad f_z \leq f_{thresh} \end{cases} \tag{S4}$$

Table S1. Optimization fitness function weight factors

w_1	w_2	w_3	w_4	w_5
10	3	2	5000	10

The fourth criterion rewards the model for making use of the assistance torque provided by AN-EXTRA-Push. This should result in minimization of total biological power across all joints:

$$f_4 = \frac{1}{t_{SimEnd}} \int_0^{t_{SimEnd}} \sum_{i=1}^{6} |P_i| \, dt \tag{S5}$$

.

We expect the biological torques during intervals of no assistance to match the trajectories observed in the NO-EXO condition. Within intervals of active assistance $[a_j, b_j]$ the changes in biological torque requirements are expected and do not affect this criterion:

$$f_5 = \frac{1}{t_{SimEnd}} (\int_0^{t_{SimEnd}} \sum_{i=1}^{6} |\tau_i - \tau_{i_{ref}}| \, dt \; - \sum_{j=1}^{4} \int_{a_j}^{b_j} \sum_{i=1}^{6} |\tau_i - \tau_{i_{ref}}| \, dt \;) \tag{S6}$$

Weight factors were chosen empirically. In healthy walking the weighted criteria should have diminishing contributions from the first to the fifth weighted criterion. Weight values are shown in Table S1. Since the optimization algorithm should minimize the fitness function, negative sign is added to the first weight (Equation S1).

Table S2. Inertial parameters of the model

Segment	mass [kg]	legnth/height [m]	COM from proximal end [m]	moment of inertia [kg m^2]
foot	1.257	0.223	0.112	0.014
toes	0.000	0.056	0.000	0.000
shank	3.369	0.398	0.172	0.049
thigh	6.600	0.421	0.182	0.122
pelvis	9.372	0.108	0.066	0.033
HAT	35.376	0.400	0.200	0.695
total body	66	1.75		

2 SUPPLEMENTARY TABLES AND FIGURES

2.1 Figures

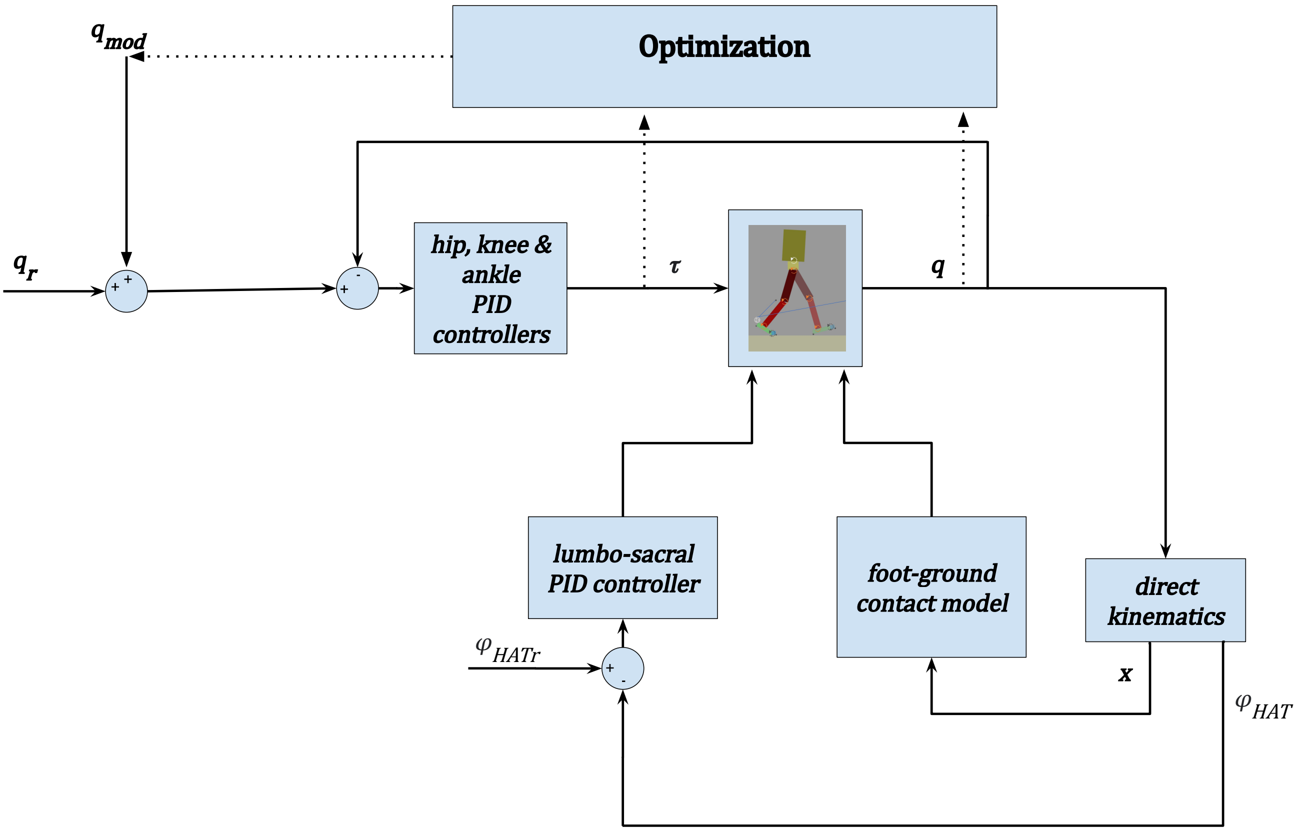

Figure S1. Dynamic walking model control scheme. Bold symbols mark vector variables. $\mathbf{q}$: joint trajectories, τ: joint torques, $\mathbf{x}$: segment positions in global coordinate system, φ_{HAT}: HAT segment orientation in global coordinate system. Optimization process produces trajectory modifications $\mathbf{q_{mod}}$ that are added to the initial reference trajectories $\mathbf{q_r}$ to create new references for local PID controllers.

2.2 Tables

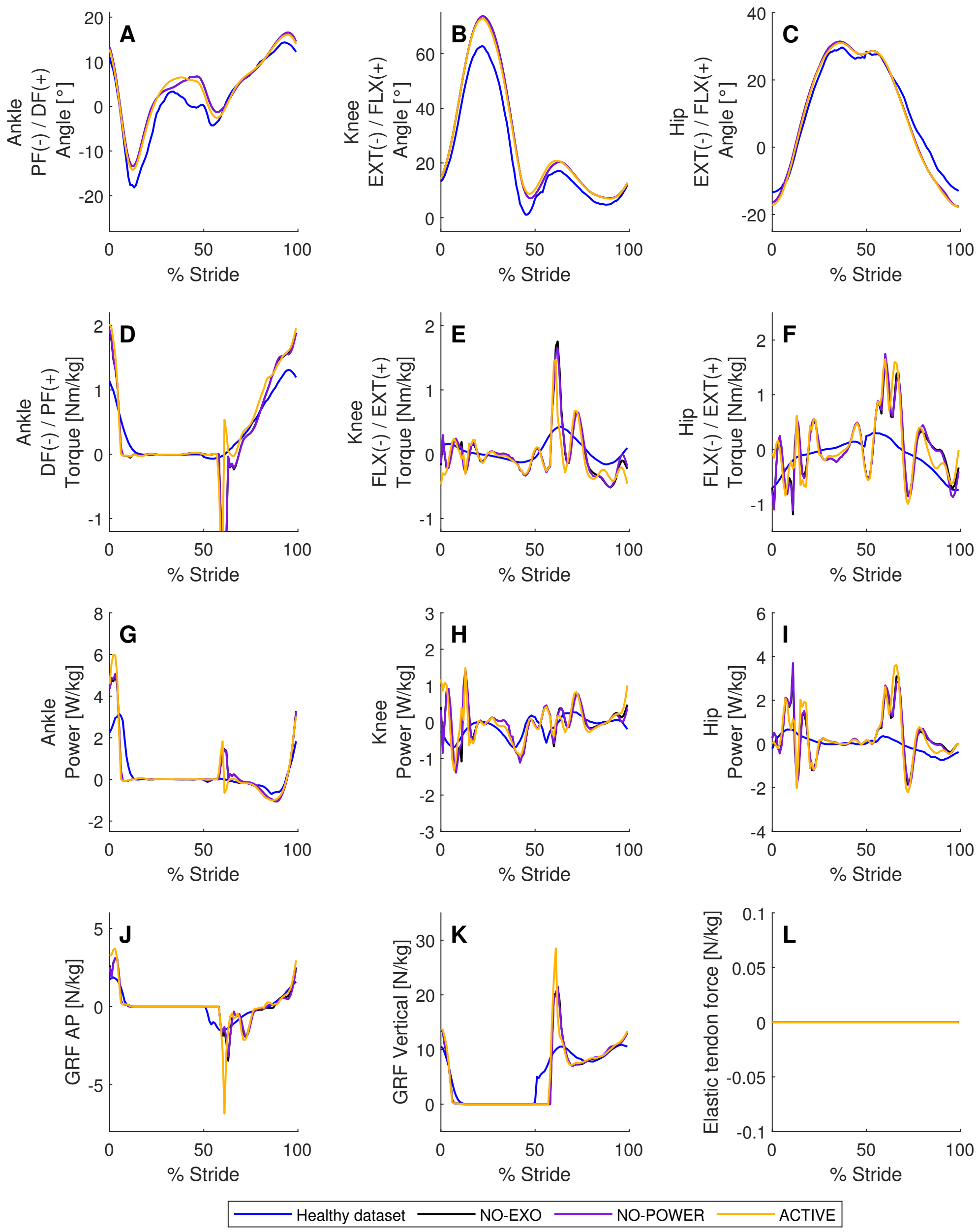

Figure S2. Comparison between a dataset of healthy people and modeled walking with an active, unpowered and without AN-EXTRA-Push exoskeleton - effects on the contralateral leg. Joint angles (**A**, **B**, **C**), biological torques (**D**, **E**, **F**) and powers (**G**, **H**, **I**) are shown for the left leg, while on the right leg AN-EXTRA-Push is worn. In **J** and **K**, antero-posterior and vertical ground reaction forces are shown. In **L**, force in the elastic tendon is shown. The stride % in abscissa corresponds to gait events of the right leg. Conditions are abbreviated and color coded as follows: healthy subjects average from a public database (Healthy dataset) (blue), without exoskeleton (NO-EXO) (black), unpowered exoskeleton (NO-POWER) (purple), and a powered exoskeleton condition with parameters: brake engagement timing of 29 % of stride, brake disengagement timing of 53 % of stride and elastic tendon stiffness of 4.85 N/m/kg (ACTIVE) (yellow).

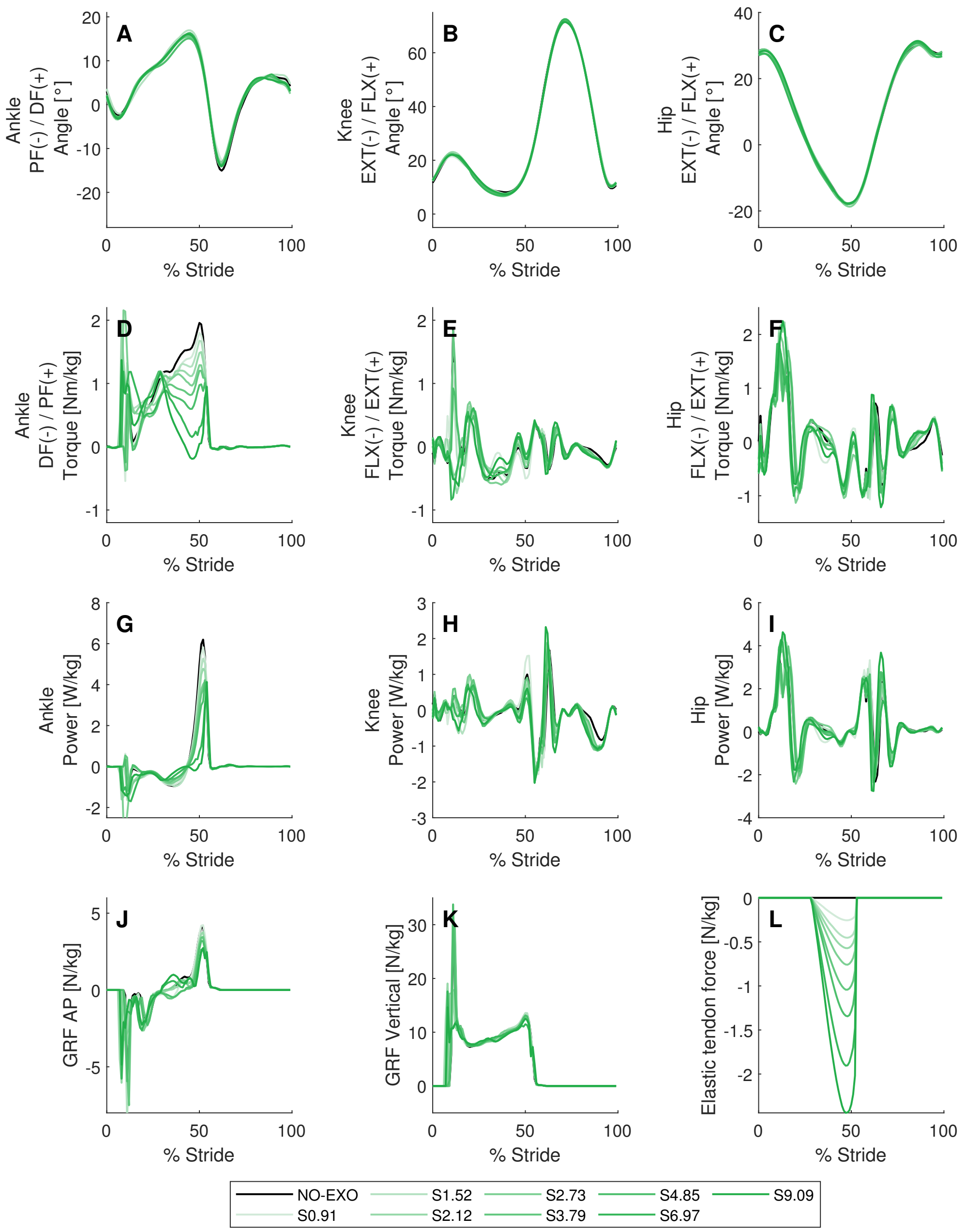

Figure S3. Elastic tendon stiffness parameter sweep supplemental figure. Joint angles (**A**, **B**, **C**), biological torques (**D**, **E**, **F**) and powers (**G**, **H**, **I**) are shown for the right leg on which AN-EXTRA-Push is worn. In **J** and **K**, antero-posterior and vertical ground reaction forces are shown. In **L**, force in the elastic tendon is shown. Brake engagement timing was set to 29 % of stride and brake disengagement timing was set to 53 % of stride. Conditions are abbreviated and color coded as follows: without exoskeleton (NO-EXO) (black) and active exoskeleton assistance with elastic tendon stiffness values of {0.91, 1.52 2.12, 2.73, 3.79, 4.85, 6.97, 9.09} N/m/kg (S0.91, S1.52 S2.12, S2.73, S3.79, S4.85, S6.97, S9.09) (green; from light to dark in the same order.)

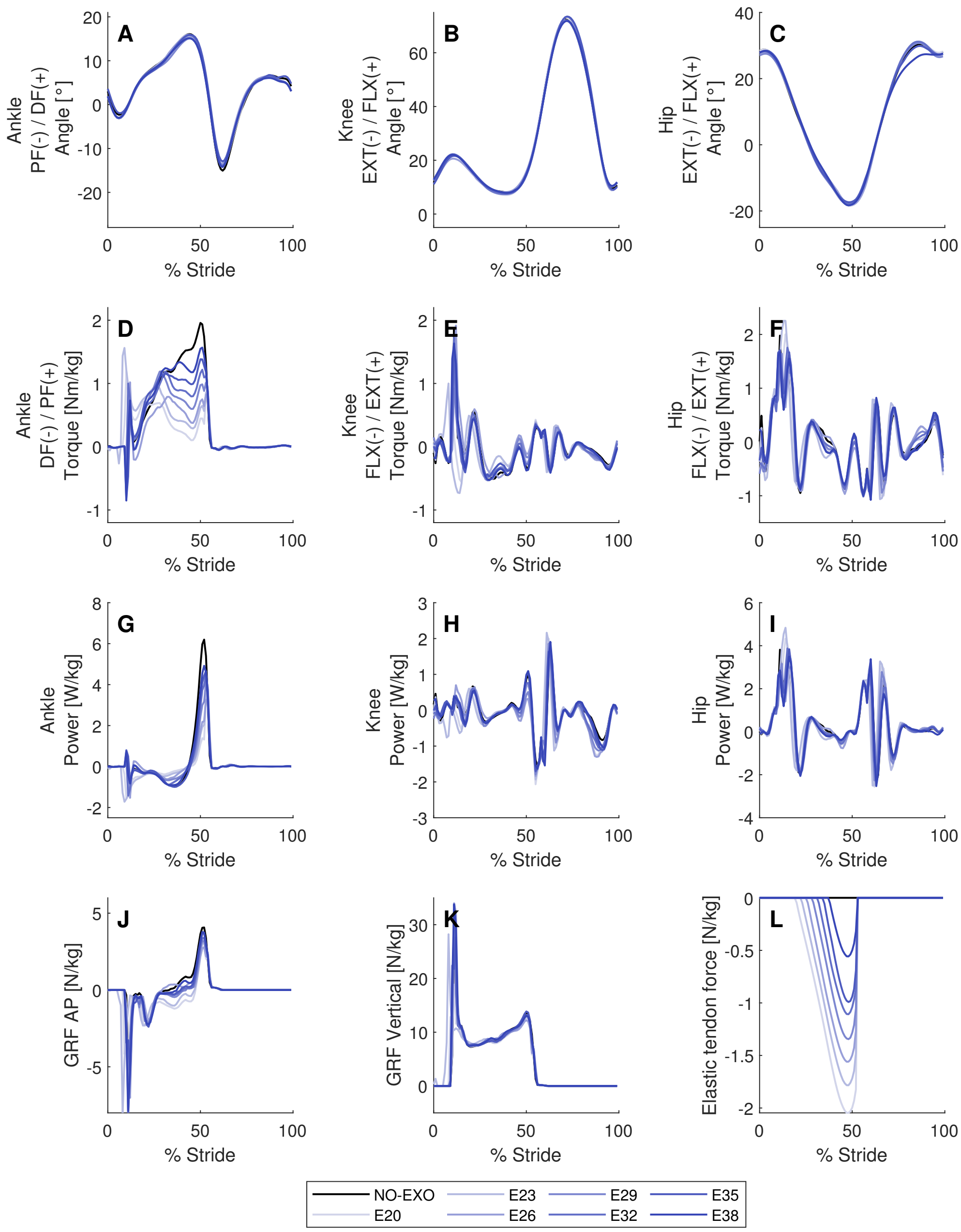

Figure S4. Brake engagement timing parameter sweep supplemental figure. Joint angles (**A**, **B**, **C**), biological torques (**D**, **E**, **F**) and powers (**G**, **H**, **I**) are shown for the right leg on which AN-EXTRA-Push is worn. In **J** and **K**, antero-posterior and vertical ground reaction forces are shown. In **L**, force in the elastic tendon is shown. Elastic tendon stiffness value was set to 4.85 N/m/kg and brake disengagement timing was set to 53 % of stride. Conditions are abbreviated and color coded as follows: without exoskeleton (NO-EXO) (black) and active exoskeleton assistance with brake engagement timings of {20, 23, 26, 29, 32, 35, 38} % of stride (E20, E23, E26, E29, E32, E35, E38) (blue; from light to dark in the same order.)

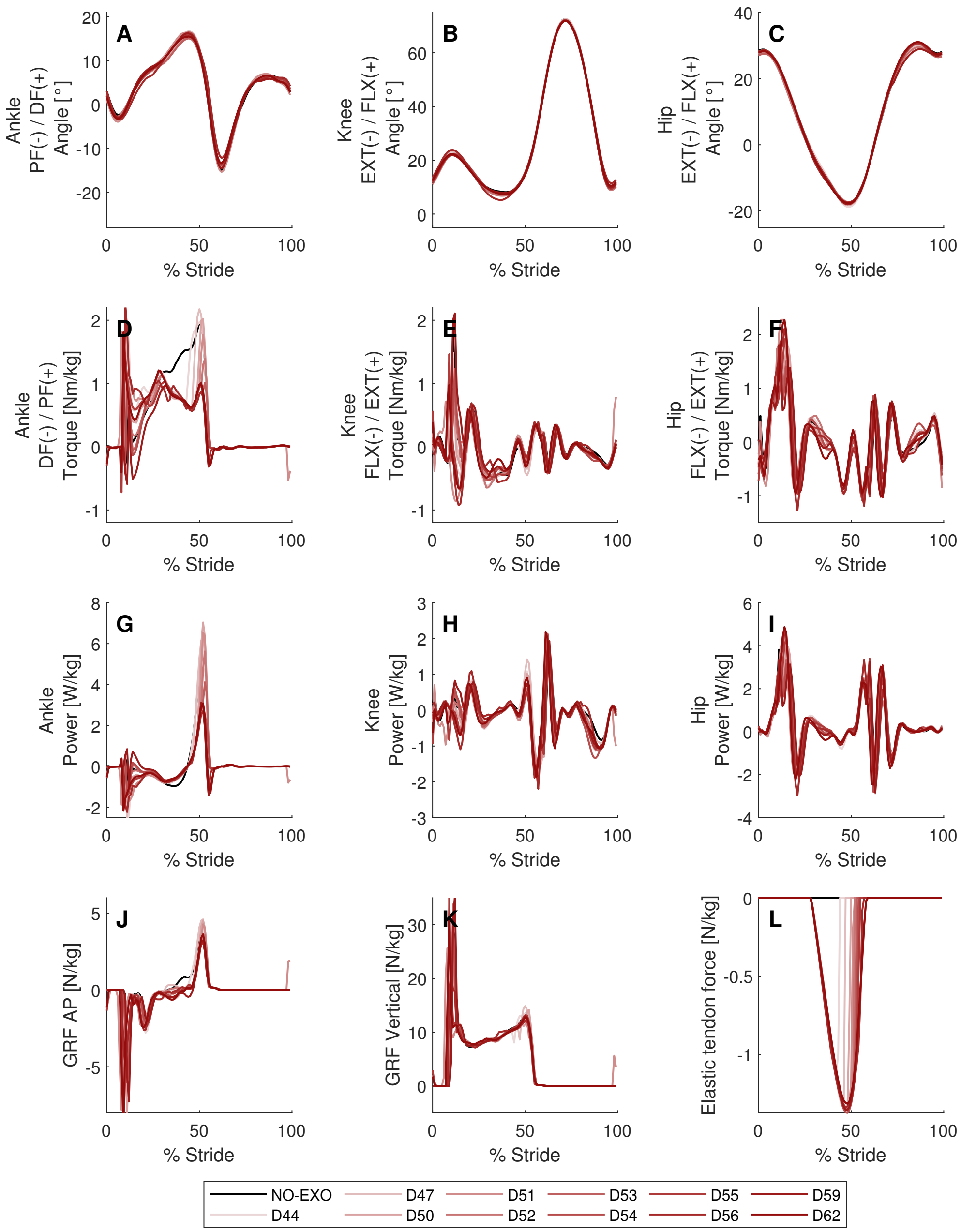

Figure S5. Brake disengagmenet timing parameter sweep supplemental figure. Joint angles (**A**, **B**, **C**), biological torques (**D**, **E**, **F**) and powers (**G**, **H**, **I**) are shown for the right leg on which AN-EXTRA-Push is worn. In **J** and **K**, antero-posterior and vertical ground reaction forces are shown. In **L**, force in the elastic tendon is shown. Elastic tendon stiffness value was set to 4.85 N/m/kg and brake engagement timing was set to 29 % of stride. Conditions are abbreviated and color coded as follows: without exoskeleton (NO-EXO) (black) and active exoskeleton assistance with brake disengagement timings of {44, 47, 50, 51, 52, 53, 54, 55, 56, 59, 62} % of stride (D44, D47, D50, D51, D52, D53, D54, D55, D56, D59, D62) (red; from light to dark in the same order.)

Table S3. Foot-ground model parameters

**Foot-ground contact parameter**	**value**
sphere radius	0.01 m
stiffness	10^5 N/m
damping	10^4 N/(m/s)
transition region width	10^{-4} m
static friction coefficient	0.95
dynamic friction coefficient	0.90
critical velocity	0.1 m/s
